# Effect of end-of-life nursing education on the knowledge and performance of nurses in the intensive care unit: a quasi-experimental study

**DOI:** 10.1186/s12912-022-00880-8

**Published:** 2022-05-03

**Authors:** Sima Sadat Ghaemizade Shushtari, Shahram Molavynejad, Mohammad Adineh, Mohsen Savaie, Asaad Sharhani

**Affiliations:** 1 Nursing Care Research Center in Chronic Diseases, School of Nursing and Midwifery, Ahvaz Jundishapur University of Medical Sciences, Ahvaz, Iran; 2 Pain Research Center, Ahvaz Jundishapur University of Medical Sciences, Ahvaz, Iran; 3 Department of Epidemiology and Biostatistics, School of Public Health, Ahvaz Jundishapur University of Medical Sciences, Ahvaz, Iran

**Keywords:** End-of-life nursing care education, Nursing education, Knowledge and performance of nurses

## Abstract

**Background:**

End-of-life care education is required for nurses to acquire the clinical competence necessary for the improvement of the quality of end-of-life nursing care. The aim of this study was to determine the effect of nursing care education based on End-of-Life Nursing Education Consortium (ELNEC) on the knowledge and performance of nurses working in the intensive care unit (ICU).

**Methods:**

This quasi-experimental study was conducted with a pretest–posttest design. From among nurses working in the ICU of Golestan and Imam Khomeini hospitals in Ahvaz, Iran, 80 nurses were selected based on the inclusion criteria. They were randomly assigned to the intervention and control groups (40 people in each group) using a table of random numbers. Data were collected using a demographic characteristics form, the ELNEC Knowledge Assessment Test (ELNEC-KAT), and the Program in Palliative Care Education and Practice Questionnaire (German Revised Version; PCEP-GR).

**Results:**

A significant difference was observed between the intervention and control groups in terms of the average knowledge score in all 9 modules including nursing care, pain management and control, disease symptom management, ethical/legal issues, culture, communication with the patient and his/her family, loss and grief, death, and quality of life (QOL) (*P* < 0.001). Moreover, the average performance score of nurses in the fields of preparation for providing palliative care, self-assessment of ability to communicate with dying patients and their relatives, self-assessment of knowledge and skills in palliative care increased significantly in the intervention group compared to the control group (*P* < 0.001).

**Conclusions:**

End-of-life nursing education is recommended as an effective method for promoting knowledge, attitude, performance, and clinical competence among all nurses involved in end-of-life care.

## Background

End-of-life care is a complex phenomenon that not only affects the patient, but also nurses and other members of the care team [1]. In this regard, many health care systems in different countries have organized end-of-life care. In addition to the care procedures for patients, end-of-life care also involves care for the patient’s family, and affects the patient’s quality of life (QOL) [1–3]. Therefore, in order to provide appropriate and effective palliative care services, nurses and other members of the health care team should receive appropriate training in end-of-life care and effective communication with patients and their family members [2, 4]. One of these programs is the End-of-Life Nursing Education Consortium (ELNEC) [5]. This program was first designed and implemented by the American Association of Critical Care Nurses (AACN) in 2001[5]. The ELNEC core course is provided in 9 modules to improve the quality of end-of-life care and to improve the vision and attitude of nurses regarding end-of-life care. These 9 modules include principles of end-of-life nursing care, pain management, sign management, ethical/legal issues, cultural considerations, communication, sorrow and mourning, attainment of end-of-life quality care, and preparation and care at the time of death [6]. The results of Glover et al. [5] indicated that participating in the ELNEC core training course improved nursing students’ knowledge about end-of-life care, especially in content areas related to palliative care, communication, symptom control, and grief-related to loss and bereavement. Sherman et al.[6] showed that, as nurses are trained in end-of-life care in undergraduate nursing curricula and through continuing education programs, it is expected that quality care will be provided, and the suffering of patients and families will be reduced.

Although the ELNEC program has been developed in the last two decades, many nurses still do not have the necessary knowledge about end-of-life care because it was not included in their educational curriculum [1]. It is recommended that end-of-life care education for nurses be included in nursing education programs [7]. In addition, the results of studies conducted in Iran have shown weaknesses in this field. In the study by Aghaei et al., nurses’ knowledge of end-of-life care was low and 55.7% of nurses stated that they had not received sufficient training in providing this care. However, the participant’s attitudes were positive toward patient care in the later stages of life [7]. Hojjati et al. found defects and deficiencies in nurses’ knowledge and attitude towards death and their nursing care of dying patients [8]. Despite the widespread holding of this course since 2001 in many countries [9], no published study or document was found in Iran on the introduction or use of a course on end-of-life nursing care as an internationally recognized course. Therefore, the researchers decided to conduct this study to assess the effect of end-of-life nursing care education based on the ELNEC on the knowledge and performance of nurses working in intensive care units (ICU).

## Methods

### Design

This quasi-experimental study was performed with a pretest–posttest design.

### Participants

This study was conducted in the ICU of Golestan and Imam Khomeini hospitals affiliated to Ahvaz Jundishapur University of Medical Sciences, Iran, from July 2019 to October 2019. Using convenience sampling method [10], 80 individuals were selected from among nurses who had the study inclusion criteria. The inclusion criteria included employment at the ICU for at least 1 year, a minimum education of bachelor’s degree in nursing, and no history of passing an ELNEC-based training course. The exclusion criteria were unwillingness to continue participating in the study or being absent in two education sessions. Due to the limited number of nurses who had the study inclusion criteria and were willing to participate in the study, random sampling was not possible and randomization was done only in the distribution of samples into intervention and control groups. The intervention type was assigned to nurses randomly using permuted block randomization with a block size of 4 (using the table on random permutation).

### Data collection tools

The data collection tools used in this study included a demographic characteristics form, the ELNEC Knowledge Assessment Test (ELNEC-KAT), and the Program in Palliative Care Education and Practice Questionnaire (German Revised Version; PCEP-GR). At first, for the translation process, the questionnaires was prepared and permissions were obtained from the developers. Two Iranian English-Persian translators independently translated the ELNEC-KAT and PCEP-GR from English to Farsi. Next, an expert panel—consisting of the research team, translators, three nursing doctorates and two experienced hospital nurses—reviewed the two translations and developed a single Farsi translation of the scales. After that, the primary Farsi version of the scales was given to thirty nurses. They were asked to comment on its readability and comprehensibility that led to minor changes based on their comments. In the present study, the reliability of ELNEC-KAT and PCEP-GR was measured as 0.86 and 0.89 (Cronbach’s α coefficient). The demographic information questionnaire including questions on age, sex, and work experience of the participants. ELNEC-KAT consists of 50 four-choice questions. The scale focuses on 9 issues related to nurses’ knowledge of end-of-life care. Each of these 50 questions has a more correct option, which is considered the correct answer [5, 11]. The PCEP-GR examines participant’s performance in end-of-life palliative care. This tool is used for the final evaluation of palliative care training courses. This scale consists of 36 items and four subscales: preparation to provide palliative care (13 question), attitudes towards palliative care (10 question), self-estimation of competence in communication with dying patients and their relatives (8 question) and self-estimation of knowledge and skills in palliative care (5 question). Each subscale is measured on a 5-point Likert-scale. The range for each subscale mean score is zero to four points, wherein higher scores indicate a higher parameter value for the respective subscale [12, 13].

### Intervention

Pretest was performed on all participants in the intervention and control groups. The intervention was performed 1 week after the pretest. The designed intervention had two parts. In the first part, nurses in charge of patient care participated in a briefing session on the importance, basics, and application of end-of-life care based on the ELNEC. The study method and stages were explained to the nurses. The intervention group patients were divided into 4 groups of 10 individuals. Next, the educational program was held for each group during five 60 and 90 min (45–70 min: training, 15–20 min: questions and answers) during 35 days (one session per week). In this course, the complete basics of end-of-life palliative care were discussed by the researcher (SSGH) based on the ELNEC. The ELNEC core course is provided in 9 modules include principles of end-of-life nursing care, pain management, symptom management, ethical issues, cultural and spiritual considerations, communication, sorrow and mourning, attainment of end-of-life quality care, and preparation and care at the time of death. The educational plan is shown in Table 1. Subsequently, the posttest was performed by the first author (SSGH) in both groups. The posttest was performed 1 month after the end of the intervention. Finally, in both parts of the tool (knowledge and performance), the score of each subscale in each test was determined and the total score of each participant was calculated in the scale through the aggregation of scores. Completion of both tools took about 60 to 70 min.

**Table 1:** The content of the educational sessions

Module	Curriculum topics	Description
Module 1	Nursing Care at the End of Life	The main concepts with regard to dying patients, the structure of providing end-of-life palliative care, providing appropriate physical and psychological space for supportive care, the role and position of nurses in the palliative care team, diagnosing a life-threatening disease and transition from the treatment stage to the palliative stage, the necessity of providing end-of-life palliative care from a human rights perspective, and gaining public satisfaction
Module 2	Pain Management	General methods of pain management, physiological, psychological and cultural/ social variables affecting pain
Module 3	Symptom Management	Managing and treating patients’ symptoms, determining the main symptoms and concerns of patients, listening actively to what patients say and paying attention to their values and beliefs, the impact of symptoms on life and physical and motor functions, and factors that aggravate or alleviate these symptoms
Module 4	Ethical/Legal Issues	Ethical/legal issues in caring for dying patients by taking into account precise cultural considerations including how to deal with a dying patient and his or her family, talking about imminent death, knowing about reasons for providing post-mortem care, continuing medical treatment, decisions as to having cardiopulmonary resuscitation, consideration of patient’s autonomy in treatment decisions, attempting to reconcile with the beliefs of the patient and his/her family, conflict resolution approaches, consideration of ethics in retelling information about diseases and death, providing spiritual care, and giving hope and comfort to the patient and his/her family
Module 5	Cultural Considerations	Evaluation of cultural and spiritual issues as a necessity for adequate communication and providing appropriate cultural care
Module 6	Communication	The need to establish effective communication in palliative care, providing support centered on the patient and his/her family, knowing the physiological, psychological, environmental and cultural barriers to communication, and making efforts to remove these barriers
Module 7	Grief, Loss, Bereavement	Discussing death, grief and bereavement after death based on cultural differences including the ways to cope with the end-of-life crisis, nursing care of the dying patient, expected physiological changes at the time of death, post-mortem care, and helping the family in adapting to the loss of life
Module 8	Achieving Quality Care at the End of Life	Focusing on care at the very time of death and emphasizing the need to be prepared and ensuring the best care while experiencing this critical incident
Module 9	Preparation and Care for the Time of Death	Identifying basic human needs at the end of life by recognizing the values, beliefs and preferences of patients, and raising knowledge of "good death"—patients’ preferences for having a good death. Finally, in addition to providing a conclusion on the material presented in the previous sessions and summarizing the topics, as is practiced in the official courses held at the End-of-Life Nursing Care Association, a case study related to end-of-life palliative care was discussed and the participants shared their own opinions. The researcher discussed each of the topics after each presentation at the end of each session. The participants exchanged their views based on their experiences in caring for dying patients

### Statistical analysis

In this study, descriptive and analytical statistical analysis methods were used in SPSS software (version 16, SPSS Inc., Chicago, IL, USA). Quantitative variables were reported as mean, standard deviation, and minimum and maximum, and qualitative variables were reported as frequency (percentage). The normality of quantitative variables was assessed using the Shapiro–Wilk test. Independent t-test, paired sample t-test and Analysis of covariance (ANCOVA) were used to data analysis. The statistical significance level was considered to be 0.05.

## Results

The mean age and work experience of the participants were 33.00 ± 4.13 and 5.26 ± 2.31 years, respectively. The number of female nurses was 75 (93.8%) and the number of male nurses was 5 (6.2%). The number of nurses with bachelor’s and master’s degrees was 73 (91.3%) and 7 (8.7%), respectively. The results of the comparison of nurse’s knowledge before and after education (the first specific goal of the study) based on the ELNEC in the intervention and control groups are presented in Table 2. There was a statistically significant difference between the intervention and control group in terms of the mean knowledge in nursing care, pain management and control, symptom management, ethical/legal issues, culture, communication with the patient and his/her family, loss and grief, death, and QOL (*P* < 0.001). The mean total knowledge score of nurses before the intervention (19.10 ± 3.91), in the intervention group, was significantly lower than the average total knowledge score of nurses after the intervention (36.69 ± 6.55) (*P* < 0.001). The highest percentage of change in the score of knowledge modules after the intervention was related to the field of death and culture.

**Table 2:** Comparison of nurse’s knowledge before and after education based on the End-of-Life Nursing Education Consortium

Module		Before the intervention	After the intervention	*P*^b^	*P*^a^
Group					
Nursing Care	Control	2.05 ± 1.41	2.00 ± 1.18	0.324	< 0.001
	Intervention	2.31 ± 1.24	4.00 ± 0.81	< 0.001	
	*P*^c^	0.397	< 0.001	-	
Pain management and control	Control	1.92 ± 1.26	2.06 ± 1.35	0.881	< 0.001
	Intervention	2.00 ± 1.12	3.94 ± 1.01	< 0.001	
	*P*^c^	0.777	< 0.001	-	
Management of disease symptoms	Control	2.65 ± 1.13	2.75 ± 1.02	0.721	< 0.001
	Intervention	2.11 ± 1.21	3.67 ± 1.43	< 0.001	
	*P*^c^	0.057	0.003	-	
Ethical/legal issues	Control	2.36 ± 1.02	2.22 ± 1.11	0.585	0.001
	Intervention	1.87 ± 1.06	3.10 ± 1.38	< 0.001	
	*P*^c^	0.045	0.004	-	
Culture	Control	2.70 ± 1.02	2.85 ± 1.32	0.839	< 0.001
	Intervention	2.26 ± 1.45	4.97 ± 1.03	< 0.001	
	*P*^c^	0.133	< 0.001	-	
Communication with the patient and his/her family	Control	2.68 ± 1.30	2.94 ± 1.11	0.130	< 0.001
	Intervention	2.22 ± 1.31	4.37 ± 1.01	< 0.001	
	*P*^c^	0.132	< 0.001	-	
Grief and loss	Control	2.71 ± 1.10	2.80 ± 1.28	> 0.99	< 0.001
	Intervention	2.55 ± 1.41	5.09 ± 1.46	< 0.001	
	*P*^c^	0.586	< 0.001	-	
Death	Control	2.05 ± 1.28	2.47 ± 1.16	0.070	< 0.001
	Intervention	1.37 ± 0.94	3.94 ± 0.88	< 0.001	
	*P*^c^	0.009	< 0.001	-	
Patient’s quality of life	Control	3.08 ± 1.19	3.17 ± 1.15	0.484	< 0.001
	Intervention	2.30 ± 1.20	4.26 ± 0.99	< 0.001	
	*P*^c^	0.005	< 0.001	-	
Total knowledge	Control	22.24 ± 4.98	24.28 ± 3.67	0.130	< 0.001
	Intervention	19.10 ± 3.91	36.69 ± 6.55	< 0.001	
	*P*^c^	0.012	< 0.001	-	

^a^Based on analysis of covariance in the presence of confounding variables (nurses’ knowledge of pre-intervention nursing care and age) has been reported

^b^Paired sample t- test

^c^Independent t-test

The results related to the second purpose of this research, which was the comparison of the performance of nurses before and after education based on ELNEC in the intervention and control groups, are presented in Table 3. There was a statistically significant difference between the control and intervention group in terms of the mean of nursing performance subscales of preparation for providing palliative care, self-assessment for the ability to communicate with dying patients and their relatives, and self-assessment of knowledge and skills in palliative care (*P* < 0.001). There was no statistically significant difference between the control and intervention group in terms of the mean performance score of nurses in the attitude towards palliative care (*P* = 0.943). Moreover, the mean performance of all nurses in the intervention group (72.15 ± 8.22) before the intervention was significantly lower than after the intervention (100.14 ± 10.00) (*P* < 0.001). The highest percentage of change in the score of performance modules after the intervention was related to preparation for providing palliative care and self-assessment of the ability to communicate with dying patients and their relatives.

**Table 3:** Comparison of nurse’s performance before and after education based on the End-of-Life Nursing Education Consortium

Module		Before the intervention	After the intervention	*P*^b^	*P*^a^
Group					
Preparations for providing palliative care	Control	26.25 ± 6.29	26.13 ± 5.57	0.557	< 0.001
	Intervention	23.77 ± 5.15	38.43 ± 4.47	< 0.001	
	*P*^c^	0.056	< 0.001	^−^	
Attitudes toward palliative care	Control	23.65 ± 3.71	24.02 ± 3.56	0.803	0.943
	Intervention	23.84 ± 2.76	23.80 ± 4.43	0.806	
	*P*^c^	0.648	0.965	^−^	
Self-assessment of ability to communicate with patients	Control	14.82 ± 4.57	15.05 ± 3.59	0.657	< 0.001
	Intervention	14.32 ± 3.42	23.28 ± 2.64	< 0.001	
	*P*^c^	0.581	< 0.001	^−^	
Self-assessment of knowledge and skills in palliative care	Control	12.00 ± 3.26	11.54 ± 3.18	0.113	< 0.001
	Intervention	10.77 ± 2.77	14.63 ± 2.35	< 0.001	
	*P*^c^	0.074	< 0.001	^−^	
Total performance of nurses	Control	76.77 ± 12.78	76.75 ± 10.44	0.478	< 0.001
	Intervention	72.15 ± 8.22	100.14 ± 10.00	< 0.001	
	*P*^c^	0.062	< 0.001	^−^	

^a^Based on analysis of covariance in the presence of confounding variables (nurses’ knowledge of pre-intervention nursing care and age) has been reported

^b^Paired sample t- test

^c^Independent t-test

## Discussion

The results of this study show a significant increase in the mean of nurse’s knowledge scores in all 9 fields of end-of-life care knowledge after the intervention. These results are consistent with the findings of Glover et al. [5]. However, the present study and above study differed in the samples studied; the study by Glover et al., was conducted on final year nursing students, while the present study was performed on nurses working in the ICU. This can be the cause of differences between the results of these two studies. One of the most important differences is the greater clinical experience of nurses working in the ICU compared to students. Undoubtedly, employed nurses have had more exposure to patients at the end of their lives and have had more experience in providing care to patients and their families. This can be effective in improving the quality of knowledge and performance. The findings of the present study indicate that participation in ELNEC training courses has increased nurses’ knowledge in all fields, especially in death and culture. However, in the study by Glover et al., the highest percentage of changes was related to palliative care, management of symptoms, communication, and elimination of grief-related loss [5]. Numerous items in the questionnaire showed that nurses had a relatively high level of prior knowledge in various fields of end-of-life care such as pain assessment and management, as well as the basic principles of nursing such as healing, suffering, and QOL of patients and their families. This may be due to the nurses’ knowledge of the general principles of nursing that they have learned during their academic studies, or due to the similarity of the educational content to the principles they have performed in sedative care at the end of life in the ICU.

In relation to the role of experience, other researchers have found that experienced nurses scored higher than less experienced nurses, meaning that nursing training and experience led to overall knowledge about palliative care [4]. These findings suggest that nurses can use the content of palliative care and end-of-life care education in the standard nursing curriculum using the ELNEC course, and any overlap in the content will have positive learning outcomes for them. Furthermore, nursing students may need to reinforce materials related to communication skills and grief in their courses [14, 15]. Previous researches have shown the nurse’s desire to become more prepared to care for dying people [16, 17]. In this regard, the need for separate courses on these topics will decrease if the content of palliative care is included in student’s curricula. The results of the present study are consistent with the results of the study conducted by Conner et al. They provided an online course on death and dying people and reported a significant improvement in nurse’s attitudes toward caring for dying people [18]. The results of the present study showed that nurses can use their knowledge of end-of-life care to promoting end-of-life care. This finding is consistent with the findings Buller et al. [19].

The second objective of the present study was to investigate the effect of education on nurse’s performance in relation to end-of-life care. Among the 4 aspects studied, the training course was found to improve the performance of ICU nurses in the 3 aspects of preparation for palliative care, self-assessment of the ability to communicate with dying patients and their relatives, and self-assessment of knowledge and skills in palliative care. Only in the field of attitudes towards palliative care, no significant improvement was observed in the nurse’s performance. This can be due to the established and stable attitudes of clinical nurses regarding their beliefs about palliative care. It seems that longer and deeper educational processes are required to induce more change in the attitudes of nurses. However, in areas in which communication and skill were desired, the ELNEC-based training course had a significant effect on improving nurse’s performance. This significant effect may indicate that short-term training courses have a more favorable effect on skills than attitudes. The results of the present study are consistent with the findings of the study by Tamaki et al. [20].

In this study, a training course was presented to improve the performance of nurses in the field of end-of-life care, and in the intervention group, improvement was observed in skills in physical performance assessment, psychological care, and confidence in providing end-of-life care. These results show that end-of-life care training enables nurses to enrich their skills in realistic situations [19]. In confirmation of the findings of the present study, Luctkar-Flude et al., reported that training courses lead to valid emotional responses to realistic patient care scenarios and lead to their imitation in real clinical conditions [21]. Heidari et al., found that posttest time is an important factor in the evaluation of training courses [22]. In this study, the researcher decided that the posttest should be implemented 1 month after the end of the training course to control the effect of the short duration of education. This is one of the important differences between the present study and some other studies in this field, including the studies by Glover et al. [5] and Tamaki et al. [20]. Further studies are required to compare several different educational strategies (both in the classroom and hospital) for end-of-life palliative care in order to identify their strengths and weaknesses. This will enable nursing educators to use the best educational approach or a combination of several strategies for nurses. In addition, it is recommended that future researches evaluate and develop interactive learning activities that improve nurse’s ability in difficult clinical projects. The first limitation of our study is that the psychometric process of the ELNEC-KAT and PCEP-GR is not performed. The second limitation is that the first author (SSGH) was involved in the course execution and data collection process, and this can lead to bias. The final limitation was that it was possible that nurses from different groups exchanged information and knowledge during their daily work, and it impacted in their performance.

## Conclusion

The ELNEC-based training course is an effective method to increase nurse’s knowledge and performance in relation to palliative and end-of-life care. Due to the growing population of the elderly in Iran, as well as around the world, courses such as ELNEC help nurses to provide the necessary and adequate supportive care to patients and their companions.

## Data Availability

Data may be available by request submitted to the corresponding author.

## Abbreviations

ELNEC: End-of-Life Nursing Education Consortium
ICU: Intensive care unit
ELNEC-KAT: End-of-Life Nursing Education Consortium Knowledge Assessment Test
PCEP-GR: Program in Palliative Care Education and Practice Questionnaire -German Revised Version
QOL: Quality of life
AACN: American Association of Critical Care Nurses
